# Effectiveness of Case Management for High-Frequency Outpatients and Long-Term Inpatients Among South Korean Medical Aid Beneficiaries

**DOI:** 10.3390/healthcare13091015

**Published:** 2025-04-28

**Authors:** Young-kyoon Na, Daho Lee, Kyounga Lee

**Affiliations:** 1 Department of Health & Welfare, Paichai University, Daejeon 35345, Republic of Korea; nayk@pcu.ac.kr; 2 Health Insurance Research Institute, National Health Insurance Service, Wonju 26464, Republic of Korea; isho@nhis.or.kr; 3 College of Nursing, Gachon University, Incheon 21936, Republic of Korea

**Keywords:** case management, Medical Aid, outpatient, inpatient, healthcare utilization, medical cost

## Abstract

**Background/Objectives:** South Korea implemented a case management program for Medical Aid (MA) beneficiaries in 2003. This study evaluates the effect of case management on healthcare utilization among MA beneficiaries, with a focus on both outpatients and inpatients. **Methods:** This retrospective comparative study was conducted using the 2023 full dataset of MA beneficiaries. The propensity score matching method was used to match the case management group with the non-case management group, and differences in healthcare utilization were analyzed using a difference-in-differences analysis. **Results:** The case management group exhibited characteristics of a medically vulnerable population, with greater healthcare needs than those of the non-case management group. Case management interventions reduced outpatient days by 4.7 and outpatient medical costs by USD 327 per person annually. For long-term inpatients, it reduced inpatient days by 13.6 and medical costs by USD 2261 per person annually (*p* < 0.001). **Conclusions:** MA case management effectively reduced both outpatient/inpatient days and medical costs. As the effects may vary depending on the type of case management, developing diverse and detailed case management programs is necessary.

## 1. Background

Rising healthcare costs have become a critical issue in many countries. In the United States, healthcare expenditure has accelerated over the past decade, with a significant increase in healthcare expenses for beneficiaries covered by federal and state-funded programs, such as Medicaid and Medicare [1,2]. Similarly, in South Korea, increasing healthcare costs for Medical Aid (MA) beneficiaries, whose healthcare expenses are funded by the government, have emerged as a major concern [3]. Total healthcare expenditure for MA beneficiaries has more than doubled over the past 10 years. As of 2023, approximately 1,524,098 individuals (approximately 3% of the total population) accounted for KRW 11.2 trillion in healthcare expenditure [4].

Although MA beneficiaries have sizeable medical needs, their use of healthcare services does not always correspond to the actual level of need [5]. Some concerns have been raised that excessive healthcare utilization may occur because MA beneficiaries either do not pay out-of-pocket costs or they pay only a minimal amount when accessing medical services [3]. The overutilization of healthcare services can lead to inefficient resource allocation and divert investments away from public health and social expenditure, ultimately placing a strain on the healthcare system. Furthermore, unnecessary medical procedures and excessive medication use may pose both physical and psychological risks to patients [6].

As structural factors contributing to the increased healthcare demand become more evident, the need to optimize and rationally manage healthcare utilization has become increasingly urgent. In this context, case management has been recognized as an effective approach [7]. Case management involves the provision of services designed to meet individuals’ healthcare needs by ensuring service quality and cost-effectiveness. Many countries have implemented case management as a strategy to enhance the quality of life of their populations while simultaneously controlling rising healthcare expenditure [8]. In the United States, a variety of case management models have been developed, particularly those targeting high-risk, high-cost patient populations. These programs highlight the necessity for well-structured designs and emphasize the need for clearly defined protocols and operational guidelines to ensure effectiveness [9]. In Canada, case management initiatives focus on frequent healthcare users with complex needs, aiming to strengthen self-management skills and enhance coordination across services [10]. Across Europe, countries such as the United Kingdom, Sweden, Germany, and Italy have adopted case management approaches tailored to their specific healthcare contexts and cultural norms. The UK tends to prioritize methodological rigor in program implementation, whereas Sweden emphasizes clarity in treatment protocols [11]. In Belgium, case management serves as a core component of integrated care for patients with chronic conditions, facilitating individualized care pathways and promoting social support interventions through multi-stakeholder collaboration [12].

In South Korea, a case management program for MA beneficiaries was introduced in 2003 to promote appropriate levels of healthcare utilization while ensuring improvements in quality of life [13]. The aim of this program is to enhance the health of MA beneficiaries and encourage rational healthcare utilization. Under this system, case managers (i.e., nurses) monitor the health status and healthcare utilization patterns of individual beneficiaries and provide tailored counseling and support [8,14]. While the program also addresses the underutilization of healthcare services among MA beneficiaries, its primary focus is preventing excessive utilization because it is funded by the national budget, which has as an aim of financial sustainability [8]. Korea’s MA case management program categorizes beneficiaries into four types: new beneficiaries, high-frequency outpatients, long-term inpatients, and year-round managed beneficiaries [14]. Among these, new beneficiaries constitute the largest group. However, as case management primarily focuses on providing guidance regarding the healthcare system, assessing any pre- and post-intervention effects resulting from its implementation is difficult. The primary target beneficiaries for managing excessive healthcare utilization are high-frequency outpatients and long-term inpatients. High-frequency outpatients are individuals who utilize medical services excessively for their health conditions. They frequently visit multiple healthcare institutions for the same condition, often resulting in duplicate prescriptions and other forms of irrational healthcare utilization. Long-term inpatients are defined as those who are hospitalized for more than 31 consecutive days for the same condition or those who undergo frequent hospital admissions and discharges exceeding one day per episode. Once designated as a high-frequency outpatient case management beneficiary, individuals receive case management interventions for three months. In contrast, long-term inpatients receive case management interventions for six months. During this period, MA case managers monitor and support patients through home visits, telephone consultations, and written correspondence to guide beneficiaries toward appropriate healthcare utilization [14]. However, despite Korea’s implementation of a national case management initiative for MA beneficiaries, there is a lack of empirical research evaluating its differential impact on distinct subgroups, such as high-frequency outpatient users and long-term inpatients.

Although individuals who frequently and/or extensively utilize healthcare services constitute a relatively small proportion of the population, they account for a significant share of total healthcare expenditure [15]. Providing case management interventions for high-frequency outpatients and long-term inpatients is therefore considered an important strategy to reduce unnecessary and excessive healthcare expenditure. Since the implementation of the MA case management program in 2003, several studies have been conducted to evaluate its impact on beneficiaries’ healthcare utilization. While many studies suggest that the program tends to reduce utilization, the results vary depending on specific outcome variables, such as the number of outpatient visits, length of hospital stay, and total medical expenses [8,16,17,18,19]. These inconsistencies might be the result of methodological limitations in previous studies, such as the failure to analyze the entire beneficiary population, absence of a control group, or lack of homogeneity within the control group, even when one was established [8,16,19,20]. Some studies did not differentiate between outpatient and inpatient case management groups based on healthcare utilization characteristics; rather, beneficiaries were analyzed as a single group [8,15]. For example, one study divided 191 high-utilization patients into intervention and non-intervention groups but found no statistically significant difference in medical expenditure between them [8]. In contrast, another study categorized 1741 case-managed individuals into outpatient, inpatient, and repeated-user groups and reported significant reductions in healthcare utilization and costs. However, it did not include a control group [16], thus limiting the interpretability of the findings and the ability to assess the net effect of each type of case management intervention. Accordingly, this study utilized comprehensive data on the entire MA beneficiary population to analyze changes in healthcare utilization before and after case management interventions. Specifically, case management beneficiaries were categorized based on their utilization patterns (high-frequency outpatients and long-term inpatients) and compared with a control group of beneficiaries who were not case-managed in order to assess differences in healthcare utilization. By addressing methodological gaps in prior studies and distinguishing between outpatient and inpatient management groups, this study aims to more rigorously evaluate the effectiveness of case management interventions by examining whether individuals who received case management experienced greater reductions in outpatient visit days, inpatient days, and associated medical costs compared to those who did not receive such interventions, thereby providing evidence to inform more targeted and efficient health policies for vulnerable populations.

## 2. Methods

### 2.1. Study Design

This study is a retrospective comparative study that examines the effectiveness of the MA case management program by distinguishing between case-managed and non-case-managed beneficiaries. Specifically, it analyzes changes in healthcare utilization (outpatient and inpatient services) by categorizing beneficiaries as high-frequency outpatients or long-term inpatients based on case management type. Given the non-randomized nature of program assignment, we employed propensity score matching (PSM) to balance baseline characteristics between groups, followed by a difference-in-differences (DID) approach to estimate the causal effect of case management while controlling for time-invariant unobserved confounders.

### 2.2. Study Sample

This study examined the entire population of MA beneficiaries in South Korea in 2023. As of that year, there were 1,659,682 MA beneficiaries, among whom 206,016 individuals (12.4%) were enrolled in the MA case management program, while 1,453,666 (87.6%) were not. The study specifically focused on two target groups: high-frequency outpatients and long-term inpatients.

To ensure a more accurate evaluation of case management effects, two subgroups of case-managed beneficiaries were excluded from the analysis. First, new beneficiaries, who accounted for approximately 70% of all case-managed individuals, were excluded because they had no prior healthcare utilization history and primarily received basic guidance regarding the MA program. Second, year-round managed beneficiaries, who used both outpatient and inpatient services extensively throughout the year, were excluded because their healthcare utilization patterns are unlikely to change within a short one-year period of case management.

As a result, the final study sample included 34,968 high-frequency outpatients and 5123 long-term inpatients who had been continuously hospitalized for 180 days prior to enrollment in the case management program. These individuals represented approximately 19.5% of all those enrolled in the MA case management program in 2023. After PSM, the final analytic sample consisted of 34,958 individuals in the high-frequency outpatient case management group and 34,958 in the matched control group, as well as 5123 in the inpatient case management group and 5123 in the matched control group.

### 2.3. Study Measures

The dependent variables in this study measured the annual utilization of healthcare services per beneficiary, including the number of outpatient visit days, outpatient medical costs, number of inpatient days, and inpatient medical costs. The annual numbers of outpatient visit days and inpatient days represented the total number of days a beneficiary received outpatient care or was hospitalized across all healthcare institutions. Outpatient and inpatient medical costs were the total expenditures incurred when a beneficiary received outpatient or inpatient treatment.

The independent variables in this study, specifically those related to the DID analysis, included group, time, and the interaction between group and time. The group variable distinguished beneficiaries who received case management intervention from those who did not. The time variable was divided into pre-and post-period. The pre-period was the one-year period before a beneficiary was enrolled in the MA case management program, whereas the post-period was the one-year period following the initiation of case management. For the control group, the pre- and post-periods were defined based on the same timeline as that of their matched case management counterparts. Additionally, a DID interaction variable was constructed to examine the interaction between group and time variables.

The control variables included demographic and socioeconomic factors, such as age, sex, and residential area (i.e., capital region, metropolitan cities, and non-metropolitan areas), as well as health-related factors that could influence healthcare utilization, including disability status and the Charlson Comorbidity Index (CCI). The CCI was used to adjust for the severity of patients’ comorbid conditions. This index assigns weighted scores ranging from 1 to 6 for 19 predefined diseases; the total score is calculated by summing the weighted values [21,22]. In this study, the CCI score was categorized into four groups: 0 points, 1 point, 2 points, and 3 or more points [23].

### 2.4. Data Collection and Ethical Considerations

This study utilized data from the National Health Insurance Service (NHIS), which maintains and manages comprehensive healthcare utilization records for MA beneficiaries. For academic and research purposes, the NHIS provides de-identified datasets to qualified researchers under strict data protection protocols. The dataset used in this study included individual-level information on demographic characteristics, eligibility for MA, enrollment in case management, and monthly healthcare utilization records, including outpatient visits, hospital admissions, and medical costs. All data were de-identified prior to analysis; individual subjects were assigned randomized temporary identification numbers to ensure anonymity. Access to the data was granted through a formal request process and was approved by the relevant data governance bodies.

The study was conducted in accordance with the principles of the Declaration of Helsinki, and a review exemption was approved by the Institutional Review Board of Seoul National University (IRB No. E-2306-033-1436).

### 2.5. Statistical Analysis

The age, sex, residential area, disability status, and CCI of MA beneficiaries were analyzed using χ^2^-tests and *t*-tests. To address the inherent selection bias in observational studies and to ensure baseline comparability between the intervention (case management) and control (non-case management) groups, we employed PSM. PSM is particularly useful in quasi-experimental designs where randomization is not feasible, as it balances observed covariates across groups, thereby approximating the conditions of a randomized controlled trial [24]. In this study, a logistic regression model was used to estimate propensity scores based on control variables, including age, sex, residential area, disability status, CCI, and healthcare utilization levels. These variables were selected based on theoretical relevance and prior empirical evidence of their influence on healthcare service use [8,16,17,18,19,20,25]. Using nearest neighbor matching, a 1:1 ratio was applied to match individuals in the case management group (high-frequency outpatients and long-term inpatients) with those in the non-case management group. After PSM, χ^2^-tests and *t*-tests were conducted to verify whether there were statistically significant differences between the case management and non-case management groups.

A DID analysis was conducted to isolate the pure effect of case management while controlling for temporal trends. DID is a robust analytical technique that controls for time-invariant unobserved heterogeneity by comparing changes in outcomes over time between the intervention and control groups. This method is particularly appropriate in policy evaluation studies, as it accounts for underlying secular trends and isolates the net effect of the intervention [26]. The DID analysis compared average differences between the case management and non-case management groups in terms of the number of outpatient visits, outpatient medical costs, number of inpatient days, and inpatient medical costs before and after case management interventions. All data analyses were performed using the SAS software (version 9.4; Cary, NC, USA).

## 3. Results

### 3.1. General Characteristics Before Propensity Score Matching

The general characteristics of the study participants before PSM are shown in Table 1. Among the 1,453,666 beneficiaries who were not in the MA case management program, the mean age was 56.2 ± 22.6 years, with 52.3% being female and 34.0% residing in non-metropolitan areas. Additionally, 15.5% had a disability and 35.1% had a CCI score of 3 or higher.

The beneficiaries designated as recipients of high-frequency outpatient case management had a significantly higher mean age of 69.9 ± 11.3 years compared to the mean age of non-case-managed beneficiaries. This population also had a higher proportion of females (64.7%) and higher percentage of people living in non-metropolitan areas (42.8%). The proportion of beneficiaries with disabilities was lower at 9.8%, whereas the percentage of those with a CCI score of 3 or higher was significantly higher at 72.7%. Before PSM, statistically significant differences were observed between the high-frequency outpatient case management group and non-case management group across all variables (*p* < 0.001).

The mean age of long-term inpatient case management beneficiaries was 71.0 ± 13.2 years. The proportion of male patients (53.5%) was higher than that of female patients. The highest proportion (49.1%) of patients resided in non-metropolitan areas. Additionally, this group had the highest percentage of beneficiaries with disabilities (29.0%), and 59.7% of the beneficiaries in this group had a CCI score of 3 or higher. Before PSM, statistically significant differences were noted between the long-term inpatient case management group and non-case management group across all variables (*p* < 0.001). As such, there were statistically significant differences in the general characteristics among the non-case management group, high-frequency outpatient case management group, and long-term inpatient case management group among MA beneficiaries in 2023.

### 3.2. General Characteristics After Propensity Score Matching

The general characteristics of the study participants after PSM are presented in Table 2. Following PSM, the final sample included 34,958 beneficiaries each in the high-frequency outpatient case management and non-case management groups and 5123 beneficiaries each in the long-term inpatient case management and non-case management groups. After matching, no statistically significant differences were found between the case management and non-case management groups in terms of sex or residential area. Although age, disability, and CCI scores remained statistically different between groups (*p* < 0.001), the discrepancies were significantly reduced compared to those before PSM. For age, the mean age of the original non-case-managed group was 56.2 ± 22.6 years. However, after PSM, the mean age of the non-case-managed group in the high-frequency outpatient category increased to 70.2 ± 13.1 years, closely matching that of the case-managed group (69.9 ± 11.3 years). Similarly, in the long-term inpatient category, the mean age of the non-case-managed group was 69.6 ± 13.5 years, comparable to the case-managed group’s 71.0 ± 13.2 years. Regarding disability status, 9.8% of individuals in the case-managed group and 10.9% in the matched non-case-managed group in the high-frequency outpatient category were registered as having a disability. In the long-term inpatient category, the proportion was 29.0% in the case-managed group and 34.0% in the non-case-managed group, indicating a well-balanced match. As for comorbidity burden, the proportion of individuals with a CCI score of 3 or higher was in the low 70% range for both groups within the high-frequency outpatient category and in the high 50% range for both groups within the long-term inpatient category.

This indicates that, following PSM, the general characteristics between the case management and non-case management groups became much more comparable within each category.

### 3.3. Effect of Case Management on Healthcare Utilization

The DID analysis results for the case management and non-case management groups after PSM matching are presented in Table 3. In the high-frequency outpatient group, the annual number of outpatient visit days per person decreased from 89.3 days before case management to 76.7 days afterward, a reduction of 12.6 days. In the non-case management group, outpatient visit days decreased from 87.3 to 79.4 days, a reduction of 7.9 days. The DID analysis indicated that case management led to an additional reduction of 4.7 outpatient visit days per person annually (*p* < 0.001). Regarding annual outpatient medical costs per person, the case management group experienced a reduction from USD 4588 to USD 4120, a decrease of USD 468. Meanwhile, the non-case management group showed a smaller reduction from USD 4771 USD to USD 4630, a decrease of USD 141. The DID analysis indicated that case management led to a further reduction of USD 327 per person (*p* < 0.001).

In the long-term inpatient group, the annual number of inpatient days per person decreased from 357.1 days before case management interventions to 303.7 days afterward, representing a reduction of 53.4 days. In the non-case management group, the number of inpatient days decreased from 361.6 days to 321.8 days, a reduction of 39.8 days. The DID analysis demonstrated that case management resulted in an additional reduction of 13.6 inpatient days per person annually (*p* < 0.001). Annual inpatient medical costs per person for the case management group decreased from USD 30,948 to USD 25,863, a reduction of USD 5085. By contrast, the non-case management group experienced a reduction from USD 29,395 to USD 26,571, a decrease of USD 2824. The DID analysis indicated that case management led to a further reduction of USD 2261 per person (*p* < 0.001).

The DID analysis confirmed that case management was associated with significantly greater reductions in both healthcare utilization and medical costs compared to the non-case management group across high-frequency outpatient and long-term inpatient populations.

## 4. Discussion

In this study, we examined the effectiveness of case management for MA beneficiaries in South Korea. Since 2003, South Korea has implemented the MA case management program in which community-based nurses provide counseling and guidance to beneficiaries exhibiting signs of excessive healthcare utilization and encourage the appropriate use of healthcare services. Based on the findings of this study, this discussion focuses on the effectiveness of case management according to the characteristics of the beneficiaries and strategies for enhancement.

First, case management programs should be designed to reflect the characteristics of MA case management beneficiaries. Compared to non-case management beneficiaries, case management beneficiaries were older and had a higher proportion of individuals residing in non-metropolitan areas rather than in the capital and metropolitan cities. Additionally, their CCI scores were higher, indicating a greater burden for comorbidities. These findings align with those of previous studies on populations who are case-managed [8], suggesting that, among MA beneficiaries, the recipients of case management interventions exhibit characteristics more closely associated with medically vulnerable populations [20,21]. With an aging population and the increasing prevalence of chronic diseases, the number of medically vulnerable individuals requiring case management is expected to increase.

Notably, the proportion of females was the highest in the high-frequency outpatient group and the lowest in the long-term inpatient group. This result is consistent with findings of previous research that categorized MA case management beneficiaries into outpatient- and inpatient-targeted groups [12]. Similarly, studies examining disparities based on sex in healthcare utilization among older adults in the United States found that women were more likely to utilize outpatient services [22,23]. This situation might be attributed to the greater healthcare needs of older women leading to more frequent outpatient visits. However, owing to limited socioeconomic resources, they may be less likely to be hospitalized than men. Women often have greater caregiving responsibilities within the household than men, and may forgo hospitalization to continue providing care at home. Additionally, they tend to prefer noninvasive treatments and procedures, which may further influence the decision to avoid hospital admission [22]. Given these findings, case managers should proactively address the healthcare needs of older women by ensuring timely interventions. For women who frequently visit outpatient clinics but who delay or avoid hospitalization, assessing whether these cases stem from disruptions in the healthcare/welfare home care system is crucial. Therefore, individualized case management for older women should focus on enhancing healthcare accessibility and coordination to promote the appropriate utilization of medical services.

Regarding disability status, the proportion of beneficiaries with disabilities was highest in the long-term inpatient case management group but lower in the outpatient case management group than in the non-case management group. This result suggests that beneficiaries with disabilities may face mobility challenges, leading to a higher likelihood of receiving inpatient care rather than outpatient-based treatment.

The higher proportion of case management beneficiaries residing in non-metropolitan areas, including rural regions, can be explained from the perspective of healthcare providers. In South Korea, the availability of healthcare resources, particularly the number of hospital beds per capita, is higher in non-metropolitan areas than in metropolitan cities [27]. Additionally, South Korea’s fee-for-service (FFS) payment system incentivizes increased healthcare utilization from the provider’s side [28]. Empirical evidence supports this trend, as the number of preventable hospitalizations has been reported to be higher in non-metropolitan areas than in metropolitan cities [27]. This finding suggests that excessive healthcare utilization may result from a combination of provider-driven factors in non-metropolitan areas and demand-side factors related to MA beneficiaries who face no out-of-pocket costs. Given that the MA case management program specifically targets high-utilization beneficiaries, strengthening regionalized case management strategies that reflect the unique characteristics of non-metropolitan areas is essential.

Second, regarding the effectiveness of case management, a comparison of outpatient/inpatient days and medical costs after PSM showed a more pronounced reduction in both days and costs within the case management group. This is the most significant finding of this study; it is partially consistent with findings of previous research, suggesting that case management is effective in reducing healthcare utilization and medical costs [8,16,25,29]. Some studies have reported that while the number of hospital visits decreased, medical costs did not decline in a corresponding manner [8]. This discrepancy could be due to differences in analytical approaches. Unlike this study, which examined outpatient and inpatient services separately, some prior studies calculated total medical expenses, including hospitalization, outpatient visits, and prescription costs. The approach used in this study, which differentiates between outpatient and inpatient services, allows for a more precise assessment of cost reduction and healthcare utilization adjustments, highlighting the importance of conducting detailed category-specific analyses of healthcare utilization when evaluating the effectiveness of case management in future research.

Since the government covers the healthcare expenditure of MA beneficiaries, excessive healthcare utilization has long been recognized as a financial burden in terms of national resources. To ensure sustainability of the healthcare system and prevent financial strain, case management must play a role in reducing unnecessary healthcare utilization. Community-based nurse-led case management can help MA beneficiaries make informed decisions and reduce overutilization through education on healthcare policies and the provision of available support systems [30]. In addition, preventive measures, including the gatekeeping role of case managers, can contribute to managing and optimizing the use of healthcare services [18]. Therefore, case management for MA beneficiaries should be continuously reinforced, not only to improve their health but also to enhance healthcare efficiency and ensure financial sustainability.

Finally, a reduction in healthcare utilization was observed in this study, not only in the case management group but also in the non-case management group, which can be interpreted as a result of the characteristics of the matched group and broader policy measures. Because the non-case management group was also matched to beneficiaries with high levels of healthcare utilization through PSM, a natural decline in utilization might have occurred following the period of intensive utilization early in an illness. In addition, the MA program has implemented various policies beyond case management to reduce excessive healthcare utilization, which might also explain the observed reduction. However, the greater reduction in both outpatient/inpatient days and medical costs in the case management group underscores the distinct impact of deliberate and structured interventions by case managers. Through counseling, health management guidance, and the coordination of appropriate healthcare use, case managers play a crucial role in actively adjusting healthcare utilization. The impact of case management is particularly significant for inpatient cases because hospitalization incurs higher medical costs than outpatient services. Thus, in the long-term inpatient group, the role of case managers in facilitating discharge and supporting the transition to home care is even more critical. These findings suggest the necessity of tailoring case management approaches based on patients’ underlying needs [31].

In South Korea, the MA case management program for inpatients extends beyond discharge planning. Case managers provide continuous support by connecting patients with community-based care services, such as home healthcare, home nursing visits, and long-term care facilities, and offer ongoing counseling and monitoring to prevent hospital readmissions [14]. Evaluating whether beneficiaries continue to receive appropriate medical care and long-term support from within the community after completing case management protocol is essential. A more structured approach to transitional care management, covering the entire process from hospitalization to discharge and home care, is needed to facilitate early discharge and successful reintegration of long-term inpatients into the community [32]. Particular attention should be paid to older adults and individuals with disabilities, who may face challenges in living independently after discharge. Strengthening community-based resources, including home nursing, home healthcare, and other care services, can create a system in which beneficiaries receive appropriate health management without relying on prolonged hospital stays.

This study has a notable strength in that it divides case management into two categories—high-frequency outpatients and long-term inpatients—and analyzes the effects of case management by extracting a control group (non-case management group) via PSM for each category. However, several limitations should be noted. First, although PSM was employed to balance the observed covariates between the case management and non-case management groups, unobserved confounding variables may still remain. Since PSM can only account for variables that are measured and included in the model, the possibility of residual selection bias due to unmeasured factors—such as unobserved patient characteristics, caregiving support, or provider-level characteristics—cannot be entirely ruled out. In addition, due to the retrospective design of the study, causal inferences should be made with caution, as the temporal ordering of events and the influence of unobserved confounders cannot be fully controlled. Second, although the DID approach helps isolate the effect of case management by controlling for time-invariant differences between groups, it assumes that the case management and control groups would have shown similar trends over time in the absence of the intervention. However, due to data limitations, we could not confirm whether the parallel trends assumption holds, which may affect the validity of the results. Third, even in the outpatient and long-term inpatient groups, variations in patient healthcare utilization patterns and disease characteristics were observed. Previous case management studies have often conducted subgroup analyses focusing on specific populations, such as emergency department patients, patients with mental health conditions, or those with alcohol use disorders [33,34,35]. Finally, the use of administrative claims data, while comprehensive, may be limited in capturing qualitative aspects of case management, such as intervention intensity, patient engagement, or contextual factors influencing health behaviors. Therefore, future research should further disaggregate MA case management groups classified by the Korean government and analyze changes in healthcare utilization based on beneficiaries’ characteristics. Additionally, as a reduction in healthcare utilization was observed in the control group (non-case management group), further research is needed to distinguish between natural decreases in utilization and the effects of case management interventions. Utilizing longitudinal data across multiple time points would allow for a more accurate assessment of these trends over time. Finally, incorporating mixed-methods designs that include qualitative insights from both beneficiaries and case managers may provide a deeper understanding of how case management influences healthcare behaviors and outcomes.

## 5. Conclusions

This study examined the characteristics of MA case management beneficiaries with excessive healthcare utilization and evaluated the effectiveness of case management. The findings indicate that case management beneficiaries represent a medically vulnerable population with greater healthcare needs than other MA beneficiaries. Case management was found to be effective in reducing outpatient and inpatient days as well as medical costs. However, the effectiveness of case management may vary depending on beneficiaries’ diseases, healthcare utilization patterns, and regional conditions. Therefore, categorizing case management beneficiaries further and developing tailored intervention strategies for both inpatient and outpatient groups is crucial for maximizing the effectiveness of case management. These findings offer several important implications for policymakers, healthcare administrators, and program designers. From a policy perspective, the results highlight the need to refine and operationalize targeting criteria for case management programs, ensuring that individuals with the highest medical and social vulnerabilities—such as those with chronic conditions, disabilities, or high healthcare dependency—are prioritized. By stratifying intervention intensity based on patient characteristics, including healthcare utilization patterns and comorbidity profiles, policymakers can more effectively allocate scarce resources and enhance the cost-efficiency of publicly funded programs. From a programmatic and practical standpoint, the study underscores the importance of developing differentiated, patient-centered intervention strategies. Tailoring case management approaches for high-frequency outpatients versus long-term inpatients can lead to more meaningful reductions in service use and improve care continuity. Furthermore, the study provides empirical support for continued investment in and refinement of case management programs as a viable policy tool for improving healthcare outcomes among medically vulnerable populations while containing overall healthcare expenditure. Strengthening the integration of case management within the broader healthcare delivery system—especially through multi-sectoral collaboration and data-driven decision making—will be critical in advancing health equity and sustainability in the public healthcare system.

## Figures and Tables

**Table 1:** General characteristics before propensity score matching.

Category		Non-Case Management	Case Management			
			High-Frequency Outpatient	χ^2^/t (*p*)	Long-Term Inpatient	χ^2^/t (*p*)
N		1,453,666	34,968	-	5123	-
Age (years) (Mean ± SD)		56.2 ± 22.6	69.9 ± 11.3	215.4 (<0.001)	71.0 ± 13.2	79.9 (<0.001)
Sex	Male	693,630 (47.7%)	12,344 (35.3%)	2110.9 (<0.001)	2741 (53.5%)	68.5 (<0.001)
	Female	760,036 (52.3%)	22,624 (64.7%)		2382 (46.5%)	
Residential area	Capital	601,273 (41.4%)	12,195 (34.9%)	1186.6 (<0.001)	1237 (24.1%)	715.7 (<0.001)
	Metropolitan	357,665 (24.6)	7808 (22.3%)		1371 (26.8%)	
	Non-metropolitan	494,728 (34.0%)	14,965 (42.8%)		2515 (49.1%)	
Disability	No	1,228,621 (84.5%)	31,537 (90.2%)	844.8 (<0.001)	3638 (71.0%)	709.9 (<0.001)
	Yes	225,045 (15.5%)	3431 (9.8%)		1485 (29.0%)	
CCI	0	426,523 (29.3%)	1360 (3.9%)	23,193.3 (<0.001)	139 (2.7%)	2175.7 (<0.001)
	1	306,059 (21.1%)	3371 (9.6%)		983 (19.2%)	
	2	210,180 (14.5%)	4825 (13.8%)		941 (18.4%)	
	3+	510,904 (35.1%)	25,412 (72.7%)		3060 (59.7%)	

CCI: Charlson Comorbidity Index.

**Table 2:** General characteristics after propensity score matching.

Category		High-Frequency Outpatient			Long-Term Inpatient		
		CM	Non-CM	χ^2^/t (*p*)	CM	Non-CM	χ^2^/t (*p*)
N		34,958	34,958	-	5123	5123	-
Age (years) (Mean ± SD)		69.9 ± 11.3	70.2 ± 13.1	−3.940 (<0.001)	71.0 ± 13.2	69.6 ± 13.5	5.221 (<0.001)
Sex	Male	12,342 (35.3%)	12,380 (35.4%)	0.090 (0.764)	2741 (53.5%)	2847 (55.6%)	4.423 (0.350)
	Female	22,616 (64.7%)	22,578 (64.6%)		2382 (46.5%)	2276 (44.4%)	
Residential area	Capital	12,195 (34.9)	12,353 (35.3)	3.391 (0.184)	1237 (24.1%)	1262 (24.6%)	3.152 (0.207)
	Metropolitan	7808 (22.3)	7618 (21.8)		1371 (26.8%)	1433 (28.0%)	
	Non-metropolitan	14,955 (42.8)	14,987 (42.9)		2515 (49.1%)	2428 (47.4%)	
Disability	No	31,527 (90.2%)	31,134 (89.1%)	23.754 (<0.001)	3638 (71.0%)	3382 (66.0%)	29.651 (<0.001)
	Yes	3431 (9.8%)	3824 (10.9%)		1485 (29.0%)	1741 (34.0%)	
CCI	0	1360 (3.9%)	916 (2.6%)	113.385 (<0.001)	139 (2.7%)	175 (3.4%)	19.399 (<0.001)
	1	3366 (9.6%)	3038 (8.7%)		983 (19.2%)	1099 (21.5%)	
	2	4824 (13.8%)	4992 (14.3%)		941 (18.4%)	996 (19.4%)	
	3+	25,408 (72.7%)	26,012 (74.4%)		3060 (59.7%)	2853 (55.7%)	

CM: Case management; CCI: Charlson Comorbidity Index.

**Table 3:** Difference-in-differences analysis results for case management and non-case management groups after PSM matching: Annual utilization per person.

Category		Group	Pre-Test	Post-Test	Post–Pre	*p*-Value
High-frequency outpatient	Outpatient visit days (Days)	CM	89.3	76.7	−12.6	<0.001
		Non-CM	87.3	79.4	−7.9	<0.001
		Difference			−4.7	<0.001
	Outpatient medical costs (USD *)	CM	4588	4120	−468	<0.001
		Non-CM	4771	4630	−141	<0.001
		Difference			−327	<0.001
Long-term inpatient	Inpatient days (Days)	CM	357.1	303.7	−53.4	<0.001
		Non-CM	361.6	321.8	−39.8	<0.001
		Difference			−13.6	<0.001
	Inpatient medical costs (USD *)	CM	30,948	25,863	−5085	<0.001
		Non-CM	29,395	26,571	−2824	<0.001
		Difference			−2261	<0.001

* USD 1 = KRW 1150; CM: case management.

## Data Availability

The research data cannot be disclosed externally. Access to the data is only available by visiting the National Health Insurance Service if required.
